# B cell intrinsic and extrinsic factors impacting memory recall responses to SRBC challenge

**DOI:** 10.3389/fimmu.2022.873886

**Published:** 2022-07-28

**Authors:** Viviana Valeri, Akhésa Sochon, Chaoliang Ye, Xinru Mao, Damiana Lecoeuche, Simon Fillatreau, Jean-Claude Weill, Claude-Agnès Reynaud, Yi Hao

**Affiliations:** ^1^ Institut Necker Enfants-Malades, INSERM U1151-CNRS UMR 8253, Université de Paris, Paris, France; ^2^ Department of Pathogen Biology, School of Basic Medicine, Tongji Medical College, Huazhong University of Science and Technology, Wuhan, China; ^3^ Department of Geriatrics, Tongji Hospital, Tongji Medical College, Huazhong University of Science and Technology, Wuhan, China

**Keywords:** germinal centers, plasma cells, GC persistence, serum antibodies, memory B cells

## Abstract

MBCs (MBCs) generated in T-dependent immune responses can persist for a lifetime and rapidly react upon secondary antigen exposure to differentiate into plasma cells (PCs) and/or to improve the affinity of their BCR through new rounds of hypermutation in germinal centers (GCs). The fate of a MBC in secondary immune reactions appears to depend upon multiple parameters, whose understanding is mandatory for the design of efficient vaccine strategies. We followed the behavior of MBCs in recall responses to SRBCs using an inducible AID fate mapping mouse model in which B cells engaged in a germinal center (GC) response are irreversibly labeled upon simultaneous tamoxifen ingestion and immunization. We used different schemes of mouse immunization and tamoxifen feeding in adoptive-transfer experiments of total splenic B cells into congenic mice that have been pre-immunized or not, to assess the contribution of the different effector subsets in a physiological competitive context. We were able to show that naive B cells can differentiate into GC B cells with kinetics similar to MBCs in the presence of previously activated T follicular helper (T_FH_) cells and a primed microenvironment. We also showed that MBCs are recruited into secondary GCs, together with naive B cells. In contrast, PC differentiation, which dominated secondary MBC responses, was not dependent upon a previous T_FH_ activation. We observed that the presence of persisting germinal centers and circulating antibody levels are key factors determining the germinal center *versus* plasma cell fate in a recall response. Notably, disruption of persistent germinal center structures by a lymphotoxin beta-receptor fusion protein or a longer timing between the prime and the boost, which correlated with reduced antigen-specific immunoglobulin levels in serum, were two conditions with an opposite impact, respectively inhibiting or promoting a GC fate for MBCs. Altogether, these studies highlight the complexity of recall responses, whose outcome varies according to immunization contexts.

## Introduction

Humoral memory response relies on two different types of lymphoid cells, long-lived plasma cells (LLPCs) and memory B cells (MBCs), which are formed during T-dependent germinal center responses (GC) where selection for improved binding affinity for the eliciting antigen takes place (1–5). LLPCs home to the bone marrow (BM), where they persist and secrete constantly high affinity antibodies (Abs) for long periods of time, but do not participate in recall responses (6). In contrast, MBCs, which emerge earlier in the GC reaction and harbor a more diverse array of Ab specificities, recirculate and can adopt different fates upon a new antigen encounter: PC differentiation through extrafollicular activation, proliferative expansion ensuring self-replenishment, or new activation in GCs for further affinity maturation, MBC and LLPC formation (7, 8). A GC-independent memory subset has also been described, formed early in the response, and displaying a major IgM isotype and low level of mutations (9, 10). The emergence of this subset has been shown to be favored by the presence of high affinity B cells within the naive B cell pool (11).

We and others have reported that IgG^+^ MBCs preferentially differentiated into short-lived PC upon a secondary challenge, while IgM^+^ MBCs had a higher propensity to adopt a germinal center fate (12, 13). Another study correlated this functional dichotomy with the MBC phenotype, describing a gradient of maturation corresponding to acquisition of CD73, CD80 and PD-L2 markers within distinct B cell subsets (14). The CD80^-^PD-L2^-^ subset, largely IgM^+^, gave rise to GC B cells upon transfer in adoptive hosts and a new challenge, while the CD80^+^PD-L2^+^ subset, mainly IgG^+^, gave rise to PC. While these different observations could appear convergent, they are not, as GC-derived MBCs have been shown to be largely CD73^+^CD80^+^PD-L2^+^, while lack of CD80 and PD-L2 expression is the hallmark of the GC-independent response (9, 15–17).

Other studies, including recent ones that were not based of MBC transfer assays, favored a recruitment of IgG^+^ MBCs in recall GCs, with B cells expressing an IgG isotype already predominating with time in the primary GC response (18), thus questioning the contribution of IgM MBCs to further affinity maturation processes (19, 20). The notion of affinity maturation as an iterative process along successive antigenic challenges was further questioned by the observation that naive B cells were by far the major subset activated in secondary GC responses (20, 21).

According to these different studies, the behavior of MBCs during recall responses remains an open question and seems to depend on different variables such as the nature of the antigen (hapten, purified protein or complex particulate antigens containing proteins and glycans), the duration of the GC reaction and the titer of antigen-specific Abs present in the circulation. A critical help for B cells in GC reactions is provided by T follicular helper (T_FH_) cells, which recruit B cells into GCs and direct their affinity maturation (22, 23). The kinetics of MBC recall responses may therefore depend also upon the presence of pre-existing T_FH_ cells (24).

To address the impact of these different factors on recall B cell responses, we used SRBCs as an antigen, and followed the behavior of GC-derived B cells though AID fate-mapping, using a reporter model (ROSA-loxP-EYFP without CAG promoter) that does not mark GC-independent activated B cells (12, 15). Different schemes of immunization were used as well as adoptive transfer experiments into congenic mice in which we could track the role of pre-existing memory T_FH_ cells on MBC responses. We report here that MBCs are mostly CD80 and PD-L2 double-positive cells, include a large IgM component and actively participate into secondary GC upon antigen recall together with naive B cells. Their involvement depends also upon several memory B cell extrinsic factors, the presence of pre-existing memory T_FH_ in the context of a primed microenvironment, long-lived GC and the waning of circulating specific Abs.

## Materials and methods

### Mouse lines

AID reporter mice were generated by breeding homozygous male AID-Cre-ERT2 x ROSA26-stop-loxP-stop-EYFP reporter mice with wt C57Bl/6 female mice and are named “AID-Cre-EYFP” throughout the manuscript. Congenic age- and sex-matched wt CD45.2 and CD45.1 (B6.SJL-*PtprcaPepcb*/BoyCrl) mice were purchased from Charles River Laboratories (France). 8- to 14-week old mice were used in this work. All experimental protocols were approved by the Ethics Committee of Paris Descartes University and validated by the French Ministry of Research.

### Immunization and tamoxifen feeding

AID-Cre-EYFP, wt CD45.2 or CD45.1 mice were immunized intraperitoneally with 1 x 10^9^ sheep red blood cells (SRBCs) (Orgentec) once, twice or three times as described in Results and Figure legends. Doses of 10 mg/mouse Novaldex (Tamoxifen; AstraZeneca) in 300 µl of 20% Clinoleic (Baxter) were administered by gavage at indicated time points after prime and boost SRBC injections.

### Lymphotoxin beta receptor-Ig fusion protein treatment

Immunized AID-Cre-EYFP mice were intravenously injected 5 times, 3 days apart, with 200 µg of lymphotoxin beta receptor-Ig (LTβR-Ig) fusion protein, purified from HEK-293 cells transfected with the corresponding expression vector by protein G binding.

### Flow cytometry analysis and cell sorting

Single cell suspensions from mouse spleens were obtained *via* mechanical disruption through 40 µm cell strainer (Falcon) and red blood cell lysis through 1xRBC Lysis Buffer (eBioscience). Cells were incubated on ice for 30 min with the combination of primary fluorochrome-conjugated antibodies or primary unconjugated biotin antibodies followed by conjugated streptavidin (complete list in **Supplementary Table S1**) in PBS supplemented with 0.5% BSA. Viability markers were also used according to manufacturer’s instructions. For intracellular isotypes staining, cells were first stained with surface antibodies then fixed and permeabilized with Cytofix/Cytoperm kit (BD Bioscience). Cells were washed, resuspended in PBS containing 2% FCS and acquired on BD LRS Fortessa cytometer (Beckton Dickinson). In cell sorting experiments, total spleen B cells were first purified with the Pan B Cell Isolation Kit II mouse (Miltenyi) following manufacturer’s instructions then sorted into GL7^+^PNA^+^EYFP^+^ GC or GL7^-^PNA^-^EYFP^+^ memory cells with a FACSAria (Beckton Dickinson). Analyses were performed with FlowJo (Tree Star Inc.) software.

### Adoptive cell transfer

Total splenocytes from AID-Cre-EYFP naive or SRBC-primed donor mice were purified through the use of the Pan B Cell Isolation Kit II mouse (Miltenyi) according to manufacturer’s instructions. Total splenocytes from wt CD45.2 naive or SRBC-primed donor mice were purified through the use of the CD4^+^ T Cell Isolation Kit mouse (Miltenyi). Where indicated, GC-depleted B cells were obtained by coupling the Germinal Center B Cell (PNA) MicroBead Kit mouse (Miltenyi) and biotin-conjugated GL7 with anti-biotin MicroBeads. IgD-biotin and anti-biotin MicroBeads mediated depletion was used before cell sorting and cell transfer in somatic mutation analysis experiments. 10x10^6^ donor cells were injected intravenously in sterile conditions in 150 µl PBS into wt CD45.1 naive or SRBC-primed mice. Recipient mice were challenged with SRBCs 2h after cell transfer and, where indicated, were fed with tamoxifen one day after. Spleens were harvested and analyzed 5 days after cell transfer.

### ELISA

Anti-SRBC IgM and IgG serum antibody titers were determined by ELISA. 96-well Nunc-immunoplates (Thermo Fisher Scientific) were coated overnight at 4°C with 1 x 10^6^/ml sonicated SRBCs and blocked with 1% BSA. Sera were diluted 500 and 10,000 times, respectively for IgM and IgG detection, and incubated 2 hours at room temperature. Goat anti-mouse IgM or IgG human-ads-HRP (Southern Biotech) were used for detection followed by KPL TMB Microwell Peroxidase Substrate System (Seracare) and colorimetric spectrophotometry at 450 and 620 nm.

### *In vitro* B cell cultures and ELISPOT assay

GL7^-^PNA^-^EYFP^+^ MBCs were sorted from a whole spleen and cultured in 6-well plates in complete RPMI-1640 medium (10% FCS, 10mM Hepes, 1X non-essential amino acids, 1mM sodium pyruvate, 5.5 x 10^-5^ M 2-mercaptoethanol, 100 U/ml penicillin, 100 µg/ml streptomycin (GIBCO)) in the presence of 3T3 40LB (as described previously) (15) with the addition of mouse rIL-4 (1ng/ml; Peprotech) for 3 days. On day 3 cells were collected and threefold cell dilutions were incubated overnight at 37°C and 5% CO_2_ in MultiScreen HTS 96-well plates (Millipore) that were previously coated overnight at 4°C with 10 µg/ml goat anti-mouse Ig (Southern Biotech) or with 1 x 10^6^/ml sonicated SRBCs and blocked with 1% BSA. After cell removal, the ELISPOT plates were incubated 1 hour at room temperature with goat anti-mouse IgM or IgG Human-ads-HRP (Southern Biotech). 3-amino-9-ethylcarbazole (BD Biosciences) was used to reveal HRP activity following manufacturer’s instruction. Red spots corresponding to individual ASCs were quantified with an ELISPOT reader using the AID software (version 3.5; AutoImmun Diagnostika) and manually counted.

### Analysis of mutations in the J_H4_ intronic sequence of the IgH locus

The intronic J_H4_ sequence flanking rearranged V_H_ gene segments was amplified by PCR from DNA of EYFP^+^ germinal center and MBC subsets from 900 up to 15,000 cells. PCR primers and reaction conditions were described previously (15). PCR products were cloned with the Zero Blunt cloning kit (Invitrogen) and sequences were determined with an ABI Prism 3130xl Genetic Analyzer. Mutations were determined within 461 bp of the J_H4_ intron through the help of the CodonCodeAligner software.

### Statistics

Results are expressed as mean ( ± SEM). Mann-Whitney test, to compare two populations or Kruskal-Wallis analysis with uncorrected Dunn’s test, for multiple comparisons, were performed to assess statistical significance with GraphPad Prism 9 Software. *p<0.05, **p<0.01, ***p<0.001, ****p<0.0001.

## Results

### Characteristics of the GC and memory response after SRBCs immunization

We studied here GC and memory recall responses against SRBC using the AID-Cre-ERT2 fate mapping mouse model. This inducible Cre line was bred with a ROSA26-stop-loxP-stop-EYFP reporter locus that lacks the CAG promoter (hereafter named AID-Cre-EYFP): this combination only allows the labeling of a limited fraction of GC B cells (see below), but AID-induced fate mapping is strictly limited to cells engaged in a GC reaction, to the exclusion of cells activated in extrafollicular responses and identified as CD38^+^GL7^+^ (**Supplementary Figure S1A**). Consequently, our model allows to track GC-derived MBCs and PCs identified as EYFP^+^B220^+^GL7^-^ and EYFP+B220^-^CD138^+^ subsets respectively (**Supplementary Figure S1B**).

**Figure 1:**
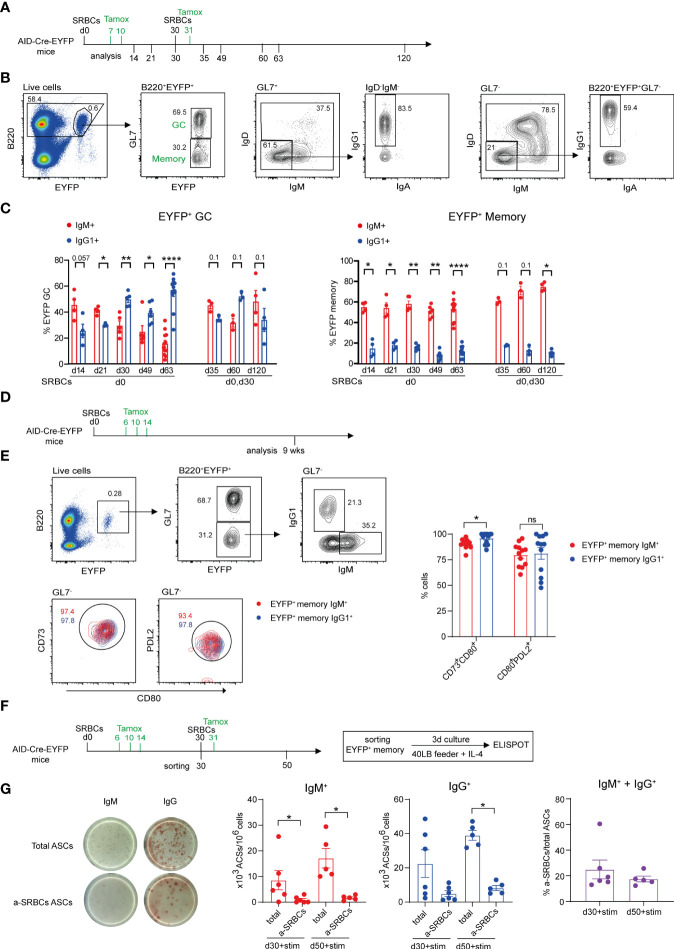
Characteristics of the GC and memory response after SRBC immunization. **(A)** AID-Cre-EYFP mice were primed i.p. with SRBCs and, where indicated, received boost injections on day 30. Tamoxifen was administrated on d7,10 and 31 (when a boost on d30 was performed). Analyses were done on splenocytes at different time points: d14, 21, 30, 35, 49, 60, 63, 120. **(B)** Representative flow cytometry gating strategy to identify GL7^+^ GC and GL7^-^ MBCs among B220^+^EYFP^+^ cells and IgM/IgD, IgG1 vs. IgA isotype subclass distribution. **(C)** Distribution of EYFP^+^ GC (on the left) and MBCs (on the right) between IgM and IgG1 isotype expression. **(D)** AID-Cre-EYFP mice immunized with SRBCs received three doses of tamoxifen on d6,10,14. Spleens were analyzed 9 weeks after prime injection to characterize maturation markers expressed by EYFP^+^ MBCs. **(E)** The expression of CD73, CD80 and PDL2 was assessed on EYFP^+^GL7^-^ IgM^+^ and IgG1^+^ memory cells. A representative flow cytometry profile is shown. Percentages of CD73^+^CD80^+^ and PDL2^+^CD80^+^ populations from IgM^+^ and IgG1^+^ EYFP^+^ memory subsets are shown in the scatter plot. **(F)** AID-Cre-EYFP mice received SRBC injection and tamoxifen gavage on d6,10,14. A group of mice was further boosted on d30 and tamoxifen fed the day after. EYFP^+^ GL7^-^PNA^-^ memory cells sorted on d30 or d50 were cultured for 3 days on 3T3 40LB feeder cells in the presence of IL-4 to induce plasma cell differentiation. To detect total and anti-SRBCs IgM^+^ and IgG^+^ antibody secreting cells (ASCs) an ELISPOT assay was conducted. **(G)** Representative ELISPOT images of IgM and IgG ASCs are shown on the left. IgM^+^ and IgG^+^ ASCs were counted and expressed as 10^3^ ASCs among 10^6^ plated cells. Percentages of IgM + IgG anti-SRBCs ASCs among total ASCs are shown in the far-right graph. Each point in the graphs represents an individual mouse. Panel **(C)** was assembled from independent experiments representing different immunization schedules, and at least two independent experiments were performed in panels **(E, G)** Means ( ± SEM) are shown. Mann Whitney test was used to compare the different conditions from panels **(C, E, (G)** Kruskal-Wallis analysis with uncorrected Dunn’s test was performed to compare the different conditions on the two graphs on the left in panel **(G)** p values>0.05 are indicated on panel **(C)**; *p<0.05, **p<0.01, ****p<0.0001. ns=not statistically significant. stim=stimulation.

It has been recently proposed that isotype switch largely takes place outside the GC reaction, while further selection shapes IgG clonal dominance during B cell proliferation in the GC environment (18, 25). We addressed the question of isotype selection in the GC reaction in the context of primary and secondary anti-SRBCs responses, and studied isotype expression on EYFP^+^ GC and MBCs at different time points, with tamoxifen given during the primary and secondary challenges (**Figure 1A**). The flow cytometry gating strategy used to determine EYFP^+^ GC and memory populations and their isotype profile is shown in **Figure 1B**. A higher proportion of IgM^+^ compared to IgG1^+^ EYFP^+^ GC B cells was identified during the first weeks of the prime immunization while IgG1^+^ cells dominated from day 30 onwards. In contrast, a more balanced isotypic profile was induced in a recall response, with equivalent IgM^+^ and IgG^+^ B cell numbers observed up to 3 months after the boost (**Figure 1C**, graph on the left). Differently from EYFP^+^ GCs, the IgM component dominated the total GC B cell response, suggesting an ongoing recruitment of naive B cells during the SRBC response (**Supplementary Figures S2A, B**). As a consequence, the EYFP labeled fraction over total GCs decreases with time (**Supplementary Figure S2C**). Throughout the primary response, EYFP^+^ MBCs were enriched in the IgM^+^ component and remained largely IgM^+^ as well upon a secondary immunization (**Figure 1C**, graph on the right). It should nevertheless be mentioned that part of the IgM^+^ EYFP^+^ MBC population originates from constant, spontaneous responses arising from endogenous antigen triggers, while the IgG1 compartment is essentially induced by the SRBC immunization (15).

The expression of the CD80 and PDL2 markers has been attributed to functionally distinct MBC subtypes with IgM^+^ and IgG1^+^ MBCs enriched in the CD80^-^PDL2^-^ and CD80^+^PDL2^+^ subsets, respectively (14). We analyzed EYFP^+^ MBCs 9 weeks after SRBCs injection and tamoxifen feeding on d6,10 and 14 (**Figure 1D**). We observed that the expression of the three markers is significantly increased on EYFP^+^ memory B cells as compared to the total B cell population and differently expressed if compared to EYFP^+^ GC B cells and that, independently of their IgM or IgG1 surface isotype, EYFP^+^ memory cells were largely enriched in double positive CD73^+^CD80^+^ and CD80^+^PDL2^+^ populations (**Supplementary Figure S3** and **Figure 1E**). These data thus confirm recent reports describing GC-derived MBCs as being essentially CD80^+^PDL2^+^ (16).

MBCs have been proposed to emerge early from the GC reaction and to be selected for low affinity antigen-binding (26). As MBC specificity cannot be assessed by direct antigen labeling in the multiple epitope context of SRBCs immunization, we determined the specificity of EYFP^+^ MBCs through an ELISPOT assay. EYFP^+^GL7^-^PNA^-^ cells were sorted 30 days after SRBC injection and 3 doses of tamoxifen feeding (d6,10,14) or 3 weeks after boost injection (d30) and additional tamoxifen feeding on day 31. Sorted EYFP^+^ memory cells were cultured for 3 days with 40LB feeder cells and IL-4 to induce MBC differentiation into PCs and the numbers of Ab secreting cells (ASCs) was assessed through ELISPOT (**Figure 1F**). As this activation promotes isotype switch in a fraction of MBCs, both IgM^+^ and IgG^+^ ASCs were considered to calculate the percentage of SRBCs-specific ASCs among activated MBCs. 20 to 25 percent of ASCs originating from EYFP^+^ MBC activation recognized SRBC antigens, a likely underestimate as not all epitopes may be correctly presented on the ELISPOT membrane (**Figure 1G**). This value nevertheless indicated that a substantial fraction of the memory pool expresses surface Ig molecules that can bind the immunizing antigen.

Bone marrow (BM) has been recently described as an important niche for MBCs (27). We thus determined the proportion of MBCs in spleen *vs.* BM 5 weeks after two immunizations with SRBCs (d0 and 30) and tamoxifen gavage on d7, 12 and 31 (**Supplementary Figure S4A**). A representative flow cytometry analysis of B220^+^EYFP^+^GL7^-^ splenic and BM MBCs and their distribution into IgM/IgD and IgG1 subsets is shown in **Supplementary Figure S4B**. In this setting, the splenic EYFP^+^ MBC population was 8 times more abundant than the one observed in the BM, and this was true for both IgM/IgD and IgG1 subsets (**Supplementary Figure S4C**). Similar numbers of B220^-^EYFP^+^ PCs were observed in the two organs at this time point (**Supplementary Figure S4D**). For all subsequent analyses, we thus focused on the splenic memory compartment, which appears to be the major one in this i.p. immunization scheme.

### Contribution of naive and antigen-experienced B cells to secondary GC reactions

To evaluate the relative contribution of naive and MBCs to secondary GCs during recall responses to SRBCs, we used an experimental setup allowing an unbiased competition between the different B cell subsets. To this end, we performed adoptive cell transfer experiments of total splenic B cells after B cell enrichment from AID-Cre-EYFP donor mice into wt CD45.1 mice.

**Figure 2:**
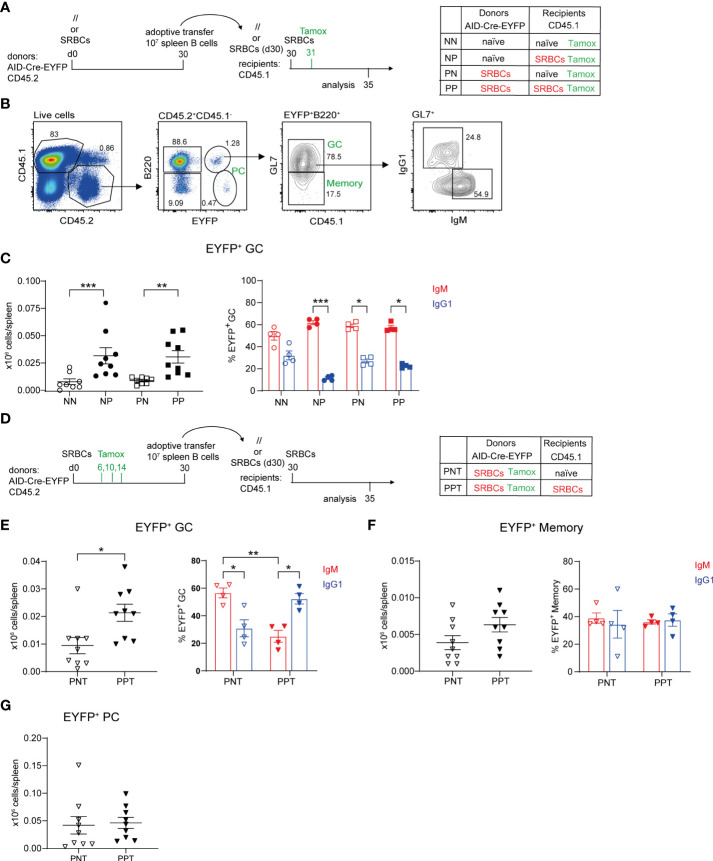
Contribution of naive and antigen-experienced B cells to secondary GC reactions. **(A)** 10 x10^6^ total purified splenic B cells from naive (//) or SRBC-primed AID-Cre-EYFP donor mice (d30) were adoptively transferred into naive or primed (d30) wt CD45.1 recipient mice. Host mice were injected with SRBCs 2 hours after adoptive cell transfer and received tamoxifen the following day. Spleens from recipient mice were analyzed 5 days after SRBC challenge. **(B)** CD45.1^-^CD45.2^+^ donor cells from recipient spleens were gated into B220^+^EYFP^+^ cells and then EYFP^+^GL7^+^ GC counts were determined, as well as their IgM^+^ and IgG1^+^ profile, and are shown in panel **(C, D)** 10 x10^6^ total purified splenic B cells from SRBC-primed AID-Cre-EYFP donor mice (d30), that received tamoxifen on d6,10,14, were adoptively transferred into naive or primed (d30) wt CD45.1 recipient mice. Host mice were injected with SRBCs 2 hours after adoptive cell transfer and spleens were analyzed 5 days after SRBC challenge. CD45.1^-^CD45.2^+^B220^+^EYFP^+^GL7^+^ GC **(E)**, CD45.1^-^CD45.2^+^ B220^+^EYFP^+^GL7^-^ memory **(F)**, and CD45.1^-^CD45.2^+^ B220^-^EYFP^-^ PC **(G)** cell numbers, as well as IgM^+^ and IgG1^+^ GC and memory subsets, are shown in the graphs. Each point in the graphs represents an individual mouse from at least two independent experiments. Means ( ± SEM) are shown. Kruskal-Wallis analysis with uncorrected Dunn’s test was performed to compare the different conditions in panel **(C)** and IgM and IgG1 analyses in panels **(E, F)** Mann Whitney test was used to compare results in panels **(E–G)** (left graphs). *p<0.05, **p<0.01, ***p<0.001. P=primed; N=naive; T=tamoxifen.

B cells were transferred from naive or SRBC immunized donors into naive or primed recipient mice, priming of recipients and donors being performed 30 days before transfer. Recipient mice were immunized with SRBCs after B cell transfer and received a tamoxifen dose the following day in order to mark GC-engaged donor B cells. Spleens from recipient mice were analyzed at d5 after cell transfer (**Figure 2A**). EYFP^+^B220^+^ cells were analyzed by flow cytometry among transferred CD45.2^+^ spleen cells and were further subdivided into GC and MBCs through the expression of GL7. IgM and IgG1 isotype expression on GC EYFP^+^ cells was also determined (**Figure 2B**). Low numbers of EYFP^+^ GC cells were obtained when B cells were transferred into naive hosts, be it from naive or primed donor mice. In contrast, when the hosts were pre-immunized, formation of EYFP^+^ GCs from both naive and primed donors were significantly increased (**Figure 2C**, left panel). No significant differences were identified between the two primed host groups, suggesting that naive B cells are engaged into secondary GC with MBC kinetics in the presence of primed T_FH_ cells. EYFP^+^ GCs were enriched in IgM^+^ cells in all conditions (**Figure 2C**, right panel).

To assess the role of memory T cells, splenic CD4^+^ T cells purified from naive or SRBC-primed (d30) wt B6 donors were adoptively transferred together with total naive purified B cells from AID-Cre-EYFP mice into naïve wt CD45.2 host mice. As in the previous setting, recipient mice were immunized with SRBCs 2 hours later and tamoxifen fed the following day (**Supplementary Figure S5A**). The subset of CXCR5^hi^PD1^hi^ T_FH_ cells identified into CD44^hi^CD62L^-^ activated CD4^+^ T cells was more abundant in SRBC-primed mice compared to naive ones. However, after CD4^+^ T cell purification, we observed a decrease of the T_FH_ subset, probably due to the downregulation of the expression of CXCR5 on these cells (**Supplementary Figures S5B–F**). Nevertheless, donor primed T_FH_ cells maintained increased expression of PD1, ICOS and CD69 memory T_FH_ associated markers before and after CD4^+^ T cell purification (**Supplementary Figures S5E, F**). EYFP^+^ GC B cell numbers were determined five days after cell transfer and immunization and a 1.6-fold increase in the GC response, albeit not reaching statistical significance, was observed in mice where B cells were co-transferred with primed-T cells compared to naive ones, supporting the hypothesis that the host participates to the faster mobilization of naive B cells (**Supplementary Figures S5G, H**). The weaker kinetic observed with this experimental setting compared to the one shown in **Figures 2A–C** may be explained by the impact of the lower CXCR5 expression in the capacity of T_FH_ to relocate rapidly to the GC environment.

Since similar GC numbers were observed after transfer of naive or primed donors, we specifically addressed the contribution of antigen-experienced B cells to recall GCs by transferring B cells from AID-fate mapped donors, and also investigated how the host status impacted their recruitment. Donor B cells from immunized AID-Cre-EYFP mice that received three doses of tamoxifen during the prime reaction (d6,10,14) were thus transferred into naive or primed CD45.1 recipients. Before adoptive transfer EYFP^+^ GCs enriched in IgG1^+^ cells accounted on average for the 30% of total GCs. EYFP^+^ MBCs were instead enriched in IgM^+^ cells. Switched IgG1^+^ EYFP^+^ MBCs represented the 10% of total IgG1^+^ MBCs, while the few EYFP^+^ PCs detectable in the spleen at this time point represented 5% of total B220^-^CD138^+^ cells (**Supplementary Figures S6A–C**). Recipient mice were immunized after cell transfer and spleen cells were analyzed 5 days later (**Figure 2D**). For antigen-experienced B cells as well, secondary GC responses were enhanced in primed hosts. Moreover, isotypic switch of antigen-experienced B cells, or recruitment of switched cells, was enhanced in T cell-help conditions (**Figure 2E**). In contrast, MBC expansion or PC differentiation of GC-experienced B cells did not require previous T_FH_ priming (**Figures 2F, G**). Differentiation of MBCs into PCs appears as the major outcome of the recall response, since EYFP^+^ PCs were 10 and 3 times more numerous than EYFP^+^ memory and GC B cells, respectively (**Figures 2E–G**).

These data clearly established that, together with naive B cells, antigen-experienced cells are recruited in secondary GCs.

### Recruitment of MBCs into recall GC responses

**Figure 3:**
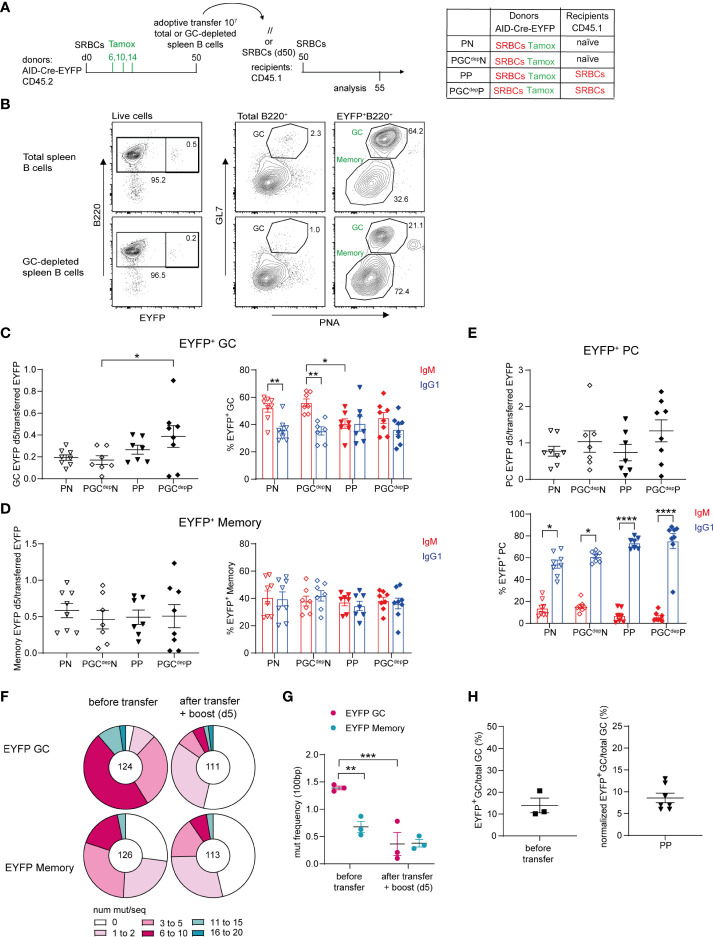
Recruitment of MBCs into recall GC responses. **(A)** 10 x10^6^ total or GC-depleted purified splenic B cells (obtained by magnetic bead depletion of PNA^+^ and GL7^+^ cells) from SRBCs-primed AID-Cre-EYFP donor mice (d50) that received tamoxifen on d6,10,14 were adoptively transferred into naive or primed (d50) wt CD45.1 recipient mice. Host mice were injected with SRBCs 2 hours after adoptive cell transfer and spleens were analyzed 5 days after SRBC challenge. **(B)** Representative flow cytometry profile of splenic cells adoptively transferred from the total B cell pool and the GC-depleted one showing the reduction of the GL7^+^PNA^+^ fraction in both total and EYFP^+^ B cells in the GC-depleted population. EYFP^+^ GC **(C)**, memory **(D)**, and PC **(E)** cell numbers (normalized on total EYFP^+^ injected cells) and their IgM and IgG1 isotype distribution are shown in the graphs. **(F)** Distribution of sequences with a given number of mutations in rearranged intronic J_H4_ sequences from EYFP^+^PNA^+^GL7^+^ GC and EYFP^+^PNA^-^GL7^-^ memory cells from AID-Cre-EYFP SRBC-primed (9 weeks) donor mice that received tamoxifen on d6,10,14 (before transfer) and from SRBC-primed (9 weeks) wt CD45.1 recipient mice that received IgD-depleted B cells from the same donors and were challenged with SRBCs after cell transfer (after transfer + boost (d5)). The total number of analyzed sequences is indicated in each corresponding pie charts. **(G)** Mutation frequencies were calculated per 100 bp and are shown in the scatter plot. **(H)** Estimate of MBC contribution to recall GCs. The left panel represents the frequency of EYFP^+^ GC B cells on total GC B cells calculated from 3 pools of donor splenic B cells before adoptive transfer (experimental setting adopted in panel **(A)**, reflecting the labeling efficiency of our reporter mouse. The right panel shows the frequency of EYFP^+^ GC counts on total GC B cells 5 days after adoptive transfer in the condition of total B cell transfer from primed donors into primed hosts (PP), after normalization for the 14% GC labelling efficiency observed after three tamoxifen gavages as calculated in the left plot. Each point in the graphs represents an individual mouse from at least two independent experiments. Means ( ± SEM) are shown. Kruskal-Wallis analysis with uncorrected Dunn’s test was performed to compare the different conditions in panels **(C–E, G)** *p<0.05, **p<0.01, ***p<0.001, ****p<0.0001. P, primed; N, naïve; GC^dep^, GC depleted.

In the previous transfer experiment performed after tamoxifen labeling, adoptive transfer of total B cells was performed to allow for the physiological competition of the different B cell subsets. However, GC B cells still represented the majority of EYFP^+^ cells 30 days after SRBC immunization. To confirm the contribution of EYFP^+^ memory B cells to secondary GCs, we transferred total B cells from immunized mice 50 days after prime (at which time point GC B cells are reduced and total and IgG1^+^ MBCs are augmented compared to d30, **Supplementary Figures S7A–C**) and added a group of donor B cells that were previously enriched in non-GC cells by negative selection on PNA and GL7-specific magnetic beads (**Figure 3A**; representative flow cytometry profile of total and EYFP^+^ B cells in **Figure 3B**).

We then assessed the number of EYFP^+^ GC B cells engaged in recall responses by comparing recipient mice that were adoptively transferred either with total or GC-depleted B cells. EYFP^+^ cell numbers observed after the boost were expressed as ratio over the total number of EYFP^+^ transferred cells, which was obviously lower in the case of GC B cell depletion. No reduction in EYFP^+^ GC cell numbers was observed upon transfer of GC-depleted B cells compared to total B cells, in both naive and primed recipient mice (**Figure 3C**, graph on the left). These results thus indicate that MBCs actively participate to secondary GCs. The same observations were made for EYFP^+^ MBCs and PCs activated in the recall response (**Figures 3D, E** graphs on the left). As observed for transfers performed at day 30, IgM^+^ cells were the main EYFP^+^ GC component in transfers into naive recipients, while comparable amounts of IgM^+^ and IgG1^+^ EYFP^+^ GCs were found in primed recipients, suggesting that isotypic switch, or switched cells, were favored by the presence of spleen memory T_FH_ cells (**Figure 3C**, graph on the right). In any case, these data clearly indicated a large contribution of the IgM memory subset to the GC recall response. IgM and IgG1 isotypes were equally represented among EYFP^+^ MBCs in the recall response, while IgG1 clearly dominated in the expanded PC population (**Figures 3D, E** graphs on the right). This suggests that switched MBCs are mostly directed to PC differentiation, even though a fraction of IgG1-expressing cells may have switched after transfer, a process that, similarly to the previous setting, was not dependent on prior T_FH_ activation (**Figure 3E**).

MBCs have been shown to harbor a lower mutation frequency in their immunoglobulin variable regions compared to contemporaneous GCs (26). We therefore analyzed the mutation frequency of EYFP^+^ GC and MBCs before and after transfer. We performed adoptive transfer of IgD-depleted splenic B cells from AID-Cre-EYFP mice primed with SRBCs 9 weeks before and tamoxifen fed at d6,10 and 14 into 9-weeks SRBCs-primed CD45.1 hosts. Recipient mice were boosted with SRBCs and analyzed 5 days later. Mutation analysis in the intronic J_H_4 sequence was performed on EYFP^+^ GL7^+^PNA^+^ GC and EYFP^+^GL7^-^PNA^-^ MBCs, sorted from a fraction of the donor cells, and the same populations were analyzed 5 days after adoptive transfer of IgD-depleted B cells (**Supplementary Figure S8** and **Figure 3F**). The mutation frequency observed for EYFP^+^ GC B cells from donor mice was 2 times higher on average compared with EYFP^+^ MBCs from the same samples (1.4 vs. 0.7, **Figure 3G**). After transfer and secondary SRBC challenge, the mutation frequency of EYFP^+^ cells engaged in secondary GCs as well as in the memory EYFP^+^ compartment displayed similar mutation frequencies, comparable to the one of the memory donor pool (**Figure 3G**). These mutation data thus confirmed that MBCs were recruited into secondary GC reactions.

To assess the relative contribution of naive and MBCs to secondary GCs, we corrected the ratio of EYFP^+^ over total GC B cells observed after transfer and re-stimulation for the labeling efficiency of our reporter mouse model. We estimated that 14% of GC B cells are labelled after three tamoxifen gavages following SRBC immunization (**Figure 3H**, graph on the left). After correcting for this labeling efficiency, a 10-fold higher contribution of naive over MBCs was thus estimated in this immunization conditions, confirming values previously reported in a different i.p. setting (20) (**Figure 3H**, graph on the right). While this proportion of MBCs among EYFP^+^ GC B cells in recall responses accounts for the lack of significant difference observed between the naive and primed donors (**Figure 2C**), the similar values observed in this setting tend also to suggest that GC-independent MBCs (present only in primed donors) are not major contributors of recall GC responses.

### The presence of persistent GCs impacts recall GC B cell responses

**Figure 4:**
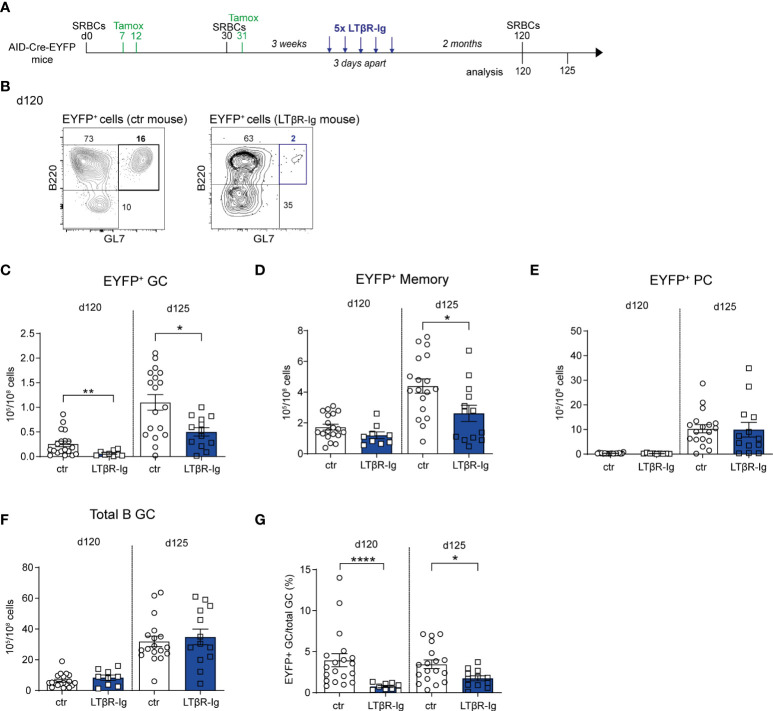
The presence of persistent GCs impacts recall GC B cell responses**. (A)** AID-Cre-EYFP mice were immunized twice with SRBCs (d0 and 30) and received tamoxifen upon prime and boost (d7, 12, 31). Three weeks after boost, a group of mice was i.v. injected with LTβR-Ig fusion protein 5 times 3 days apart. Spleen cells from treated and control (ctr) mice (that were immunized similarly without LTβR-Ig injection) were analyzed by flow cytometry before (d120) or 5 days after (d125) a tertiary SRBC injection performed on d120. **(B)** Representative flow cytometry plots showing the depletion of EYFP^+^GL7^+^ GC B cells in LTβR-Ig-treated mice on d120. EYFP^+^ GC **(C)**, memory **(D)**, PC **(E)**, and total B GC **(F)** cell numbers are shown for control and treated mice on both d120 and 125. The fraction of EYFP^+^ GCs among total GC B cells is shown in panel **(G)** Each point in the graphs represents an individual mouse from at least two independent experiments. Means ( ± SEM) are shown. Mann Whitney test was used to compare control and treated mice at the two time points analyzed. *p<0.05, **p<0.01, ****p<0.0001. ctr=control mice.

SRBC immunization induces GC reactions that last over several months, a process likely contributed by the persistence of antigen deposits on follicular dendritic cells (FDCs) (12). The FDC network is maintained through the provision by B cells of lymphotoxin (LTαβ), acting on the LTβ receptor it expresses (28). We assessed the role of persistent GCs on GC recall responses by interfering with the LTαβ-LTβR axis. To this end, AID-Cre-EYFP mice were primed and boosted with SRBCs (day 0 and 30) and received tamoxifen on d7, 12 and 31; they were injected 5 times with LTβR-Ig, 3 weeks after the SRBC boost to deprive the B cell-FDC interaction and promote GC disruption (**Figure 4A**; representative flow cytometry plot showing B220^+^EYFP^+^GL7^+^ GC depletion 60 days after LTβR-Ig treatment on **Figure 4B**). A tertiary immunization was performed at day 120, 60 days after LTβR-Ig treatment, to allow for the FDC network to reconstitute. EYFP^+^ GC B cell numbers before the third boost were significantly reduced in spleen of LTβR-Ig treated mice compared to control mice while EYFP^+^ memory and PC numbers remained unchanged (**Figures 4C–E**). After the third immunization (d125), lower EYFP^+^ GC and MBC numbers were observed in mice that received the LTβR-Ig treatment compared to controls (**Figures 4C, D**). Persistent and recalled IgG1^+^ EYFP^+^ GCs were more specifically affected by the treatment while both IgM^+^ and IgG1^+^ EYFP^+^ memory cells were reduced upon boost (**Supplementary Figures S9A, B**). The total PC response was not impacted upon recall (**Figure 4E** and **Supplementary Figure S9C**). Differently from EYFP^+^ GC B cells, the total GC response upon boost immunization was not affected by the treatment, suggesting a different impact on naive vs. MBC recruitment (**Figures 4F, G**). Altogether these results suggest that GC and MBC recall responses are affected when interfering with the persistence of GCs. For GC B cells, this could result from a decreased MBC recruitment or a decreased activation of GC B cells, these two non-mutually exclusive scenarios leading to a decreased proportion of antigen-experienced over naive B cells in recall GCs.

### Longer time elapsed between prime and boost favors GC and MBC recall responses

**Figure 5:**
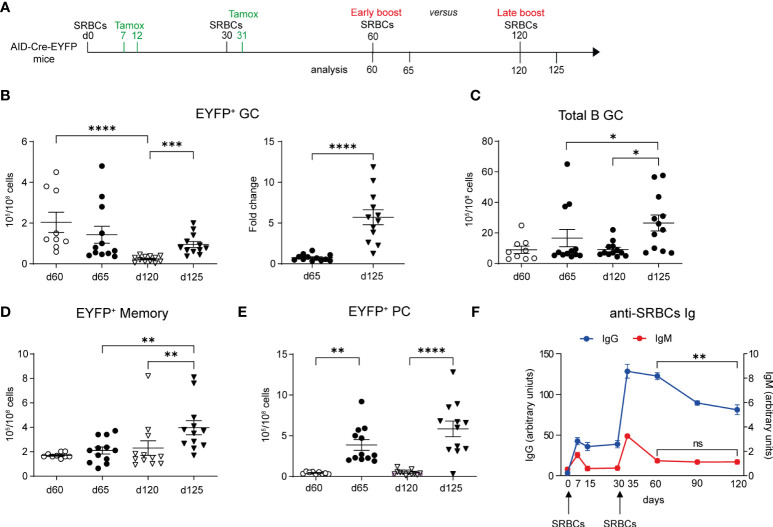
Longer time between prime and boost favors GC and memory recall responses. **(A)** AID-Cre-EYFP mice were immunized twice with SRBCs (d0 and 30) and received tamoxifen upon prime and boost (d7, 12, 31). Spleen cells were analyzed by flow cytometry on d60 or 120. Alternatively, mice received a third early or late SRBC boost at, respectively, d60 or 120 and were analyzed at d65 or 125. **(B)** EYFP^+^ GC cell numbers are shown in the left graph for both boost schedules. On the right, the fold change of EYFP^+^ GC cell numbers on d65 and 125 related to the average cell number observed before the early and late boosts is shown. Numbers of total B220^+^GL7^+^ GC B cells **(C)**, EYFP^+^ memory **(D)** and PCs **(E)** are shown in the corresponding graphs. **(F)** anti-SRBC IgM and IgG titers, represented as arbitrary units, were determined by ELISA from serum of mice that received SRBC injections on d0 and 30. Curves are obtained by analyzing serum collected longitudinally from 6 mice from d0 until d120. Each point in the graphs represents an individual mouse from at least two independent experiments **(B–E)**. Means ( ± SEM) are shown. Kruskal-Wallis analysis with uncorrected Dunn’s test was performed to compare the different conditions in panels **(B)** (graph on the left)**–E**. Mann Whitney test was used to compare results in panel **(B)** (graph on the right) and d60 vs 120 from graph in panel **(F)** *p<0.05, **p<0.01, ***p<0.001, ****p<0.0001. ns=not statistically significant.

We next addressed the impact of the boost timing on recall B cell responses, an important issue for tertiary vaccine challenges. We performed a tertiary SRBCs immunization in which mice received either an early or a late boost, respectively on d60 or d120, and analyzed GC and MBC responses 5d after (**Figure 5A**). Upon an early boost, EYFP^+^ GCs numbers were not different from the ones observed before the third immunization. In contrast, EYFP^+^ GC B cell numbers significantly increased 5 days after a late boost with respect to numbers observed at d120 (**Figure 5B**, graph on the left). The fold change of EYFP^+^ GC B cell numbers upon a late boost was indeed 5 times higher than the one observed upon an early boost (**Figure 5B**, graph on the right). By looking at the total GC B cell population that contains also naïve B cells engaged into tertiary GCs upon boost, we similarly observed that the whole GC B cell response was more effective in a late boost strategy (**Figure 5C**). Similar results were obtained for EYFP^+^ memory cells that efficiently expanded only when the third immunization was performed at late time points (**Figure 5D**). For the PC response, similar numbers of EYFP^+^ PCs were observed in both boost timings (**Figure 5E**).

A competition for the B cell receptor ability to capture the antigen and for further differentiation in the presence of high levels of antigen-specific circulating Abs has been proposed (13). We therefore followed the level of serum anti-SRBC Abs over time (d0 to d120) for mice immunized and boosted at day 0 and 30. We observed a significant decrease in serum IgG1 level between d60 and d120, while IgM levels were already close to baseline at day 60 (**Figure 5F**). Better recall MBC responses may thus be correlated with the decay of circulating Ag-specific Abs.

## Discussion

We profiled in this study the recall response to SRBC, a complex antigen with large epitope diversity, and analyzed different parameters, T cell help, persistent GCs and timing of booster immunization for their impact on recall GC formation, MBC expansion and PC differentiation. We used the AID-Cre-EYFP reporter line to fate map GC-derived MBCs, a model in which, due to the limited accessibility of the ROSA26 locus, only GC B cells and not B cells derived from extrafollicular responses are marked in the course of the immune response.

We first analyzed the contribution of naive and MBCs to recall GC responses by performing transfer of total splenic B cells from immunized or non-immunized AID-Cre-EYFP donors into immunized and non-immunized wildtype hosts, a setting that allows for physiological competition of the different B cell subsets in the secondary response. The contribution of naive B cells was inferred from transfer experiments in which AID-mediated labeling was performed one day after transfer, while information about MBCs was obtained from experiments using cells labeled before transfer. In the first setting, we observed that naive B cells can be recruited into GC with kinetics similar to MBCs only in the case of a pre-immunized host, clearly showing that memory T cell help is instrumental in such rapid mobilization in the context of a primed microenvironment. Interestingly, this situation may pertain to cases of successive infections with distantly related pathogens, which could harbor B-cell neo-epitopes but still conserve peptides that elicit memory T_FH_, as observed between seasonal coronaviruses and SARS-CoV-2 (29, 30). Whether this early engaged subset of naive B cells also includes a “pre-GC/naive-like” population that may have a competitive advantage compared to “more naive” B cells remains to be determined.

Using transfer of total B cells labeled during pre-immunization, we also confirmed the mobilization of MBCs in recall GCs, a response that was similarly affected by pre-existing T_H_ cells. In contrast, differentiation of MBCs into PC appears largely independent of T cell help, with no quantitative difference observed according to the status of the host. Such T-independent differentiation has been reported for anti-viral responses, in a complex setting of transfer into RAG-deficient mice (31).

Following SRBC immunization, the EYFP^+^ MBC pool comprises a large IgM memory compartment, together with IgG1 MBCs, and harbors for its vast majority a CD73^+^CD80^+^PD-L2^+^ profile, similar for both isotypes. These data confirm several reports indicating that the lack of expression of these markers is the hallmark of B cells activated outside germinal centers (9, 16), and therefore do not distinguish maturation stages within the MBC pool. After transfer of B cells from pre-immunized mice and AID-mediated labeling, an important population of IgM^+^ cells were observed in recall GCs, albeit less predominant in a pre-immunized host, suggesting that memory T helper cells favor either the recruitment of IgG1^+^ precursors, or their rapid isotype switch before differentiation into GC B cells, or both. Interestingly, while IgG1^+^ cells tend to dominate over IgM^+^ after 4 weeks of the GC response, isotype distribution appears more equilibrated in secondary GCs up to 3 months after challenge, a configuration in adequation with the isotype profile of the memory pool they feed, and thus not reflecting a dominant mechanism of positive selection of IgG^+^ over IgM^+^ cells in GC reactions as proposed recently (18). In all cases, PC differentiation was the predominant event in terms of cell expansion during recall responses, with a major IgG1 component, 5 days after secondary challenge. However, despite PCs being numerically superior, there was no exhaustion of the MBC population after recall immunizations.

We estimated that MBCs contributed to approximately 10% of the GC recall response, with naive B cells thus dominating in secondary challenges. A still lower contribution of MBCs to recall GCs was reported in the setting of a boost in the contra-lateral footpad, but values similar to ours were mentioned in this study for an intraperitoneal immunization (20). This is in line with a recent paper which shows the preferential participation of MBCs to recall GCs in local boost condition (32). In the case of an SRBCs challenge that generates robust secondary GCs comprising several millions of cells, the contribution of MBCs, even minor, still represents a considerable cell fraction that will undergo new rounds of maturation. Synchronous recruitment of naive and MBCs into GC was also reported in humans, through fine needle puncture of draining lymph nodes following flu vaccination, but in variable proportions among patients (33). However, the proportion of these recalled clones was on average higher than what observed in mice, suggesting that recall MBC responses in humans may be more important than what observed in mouse models. Which parameters, including notably the amount of T cell help, may modulate the relative contribution of naive vs. MBCs to recall responses remains to be addressed.

The SRBC immunization setting does not provide adequate conditions for tracking antigen-specific MBCs or analyze their affinity, due to the antigenic complexity this immunogen represents. We nevertheless estimated that 20-25% of MBCs, after both primary or secondary challenges, show specificity against SRBCs in a memory ELISPOT assay. This proportion is obviously an under-estimate, as not all antigens that elicited a memory response may be properly displayed in the sonicated red blood cell lysate. This assay nevertheless indicates that a consistent fraction of GC-derived MBCs display clear antigen specificity, in contrast to a recent report that similarly analyzed fate-mapped MBCs (26).

SRBC immunization generates persistent, residual GC structures, in which EYFP^+^ GC B cells can be observed up to 8 months following a secondary challenge (12). This configuration, which differs from the one observed after protein immunization (e.g. hapten-carrier in Alum), is likely caused by the lower biodegradability of this particulate antigen comprising numerous glycoproteins, allowing their persistence within the FDC network. To interfere with these long-lasting GC structures, we repeatedly injected LTβR-Ig to block the crosstalk between GC B cells and FDCs through the LTαβ/LTβR pathway, after two SRBCs immunizations. GC persistence was strongly reduced 2 months after this episode of LTαβ sequestering, and tertiary SRBC challenge resulted in a lower recruitment of recall GC B cells while naive B cells were recruited normally. This experiment therefore suggests a difference in the mobilization of naive and memory B cells and that the existence of residual GC structures may favor the rapid mobilization of MBCs and/or amplification of residual GCs, both antigen-experienced subsets and the restart of the GC reaction.

The timing of booster immunization is a major issue in setting vaccination schedules, and previous data have suggested that antigen-specific serum IgG, either pre-existing or produced through rapid differentiation of IgG-secreting ASCs following secondary challenges, may impact the mobilization of MBCs into GCs (13). When longer elapsed time was allowed between secondary and tertiary challenges (from 1 to 3 months), recruitment of MBCs into recall GCs as well as expansion of the memory pool were enhanced, but their differentiation into ASCs was only marginally impacted. These different behaviors correlated with a time-dependent waning of SRBC-specific IgG levels in mouse serum, suggesting that circulating antibodies may differentially impact activation pathways leading to GC recruitment and MBC expansion compared to PC differentiation.

Altogether, recall responses to SRBC challenges present several features that differ from some other models of immune responses, like those based on protein or hapten-carrier immunizations, notably in terms of isotype distribution during the recall GC reaction and within the memory pool, as well as for MBC antigen specificity. Distinct memory profiles, harboring notably a large IgM component, have indeed been reported in immune responses against parasites or viruses (34–36). It will be obviously important to understand whether these features pertain more generally to the immune response to whole pathogens, which may share biochemical features with SRBCs.

## Data availability statement

The raw data supporting the conclusions of this article will be made available by the authors, without undue reservation.

## Ethics statement

The animal study was reviewed and approved by the Ethics Committee of Paris Descartes University and by the French Ministry of Research.

## Author contributions

YH, VV, J-CW and C-AR designed the experiments. YH, VV and AS performed most of the experiments, together with CY, XM and DL. VV, J-CW and C-AR wrote the first draft of the paper. All the authors critically reviewed the manuscript. All authors contributed to the article and approved the submitted version.

## Funding

This work was supported by the ERC Advanced grant “B-response”, by the Fondation Princeasse Grace de Monaco, by the Fundamental Research Fund for the Central Universities (HUst:2015ZHYX007) and by The International Cooperation Base of Hubei Province for Infection and Immunity.

## Acknowledgments

The authors thank the expert assistance of the core facilities of the Structure Fédérative de Recherche Necker (INSERM US24/CNRS UMS5633), in particular Pierre Chérel, Jean-Christophe Bèche and Virginie Caulet from the animal facility and Jérôme Mégret from the Cytometry platform.

## Conflict of interest

J-CW received consulting fees from Institut Mérieux, outside of the submitted work.

The remaining authors declare that the research was conducted in the absence of any commercial or financial relationships that could be construed as a potential conflict of interest.

## Publisher’s note

All claims expressed in this article are solely those of the authors and do not necessarily represent those of their affiliated organizations, or those of the publisher, the editors and the reviewers. Any product that may be evaluated in this article, or claim that may be made by its manufacturer, is not guaranteed or endorsed by the publisher.
