# Supplementary material for: B cell intrinsic and extrinsic factors impacting memory recall responses to SRBC challenge
Source: Front Immunol. 2022 Jul 28;13:873886. doi: 10.3389/fimmu.2022.873886 (PMC9367638; doi:10.3389/fimmu.2022.873886)
Supplement: Supplementary file 1 [file DataSheet_1.pdf]

B cell intrinsic and extrinsic factors impacting memory recall responses to SRBC challenge

Viviana Valeri[1*], Akhésa Sochon[1], Chaoliang Ye[2], Xinru Mao[2], Damiana Lecoeuche[1], Simon Fillatreau[1], Jean-Claude Weill[1], Claude-Agnès Reynaud[1], Yi Hao[2,3*]

[1]Institut Necker Enfants-Malades, INSERM U1151-CNRS UMR 8253, Université de Paris, 156-160 rue de Vaugirard - 75015 Paris, France
[2]Department of Pathogen Biology, School of Basic Medicine, Tongji Medical College, Huazhong University of Science and Technology, Wuhan, China
[3]Department of Geriatrics, Tongji Hospital, Tongji Medical College, Huazhong University of Science and Technology, Wuhan, Hubei, China

*** Correspondence:**
Corresponding Authors: viviana.valeri@inserm.fr and haoyi@hust.edu.cn

Supplementary Table S1. List of antibodies and reagents used in the work.

Antibodies	Clone	Manufacturer	Catalog number	Batch
Anti-mouse B220	APC-efluor780, clone RA3-6B2	eBioscience	47-0452-82	2272766
Anti-mouse B220	PE-efluor610, clone RA3-6B2	eBioscience	61-0452-82	2331160
Anti-mouse CCR6	PE, clone 29-2L17	Biolegend	129804	B257642
Anti-mouse CD38	PerCP-Cy5.5	Biolegend	102721	
Anti-mouse CD138	PE-Cy7, clone 281-2	Biolegend	142514	B291316
Anti-mouse CD38	Biotin, clone 90	BD Biosciences	553762	78384
Anti-mouse CD45.1	Efluor450, clone A20	eBioscience	48-0453-80	2187619
Anti-mouse CD45.2	PE, clone 104	BD Bioscience	560695	8198750
Anti-mouse CD73	PE-Cy7, clone eBioTY/11.8	eBioscience	25-0731-82	2016450
Anti-mouse CD73	Efluor450, clone eBioTY/11.8	eBioscience	48-0731-82	4314359
Anti-mouse CD80	APC, clone 16-10A1	Biolegend	104714	B227918
Anti-mouse CD80	PE, clone 16-10A1	Biolegend	104707	B2982893
Anti-mouse CD80	BV510, clone 16-10A1	Biolegend	104741	B331397
Anti-mouse GL7	Efluor450, clone GL7	eBioscience	48-5902-82	2062737
Anti-mouse GL7	PerCP-Cy5.5, clone GL7	Biolegend	144610	B326932
Anti-mouse PNA	Biotin	Vector Laboratories	B-1075	ZD1101
Anti-mouse IgA	PE, clone 11-44-2	eBioscience	12-5994-81	2252069
Anti-mouse IgA	Biotin	Sony	2635015	155924
Anti-mouse IgD	PerCP-Cy5.5, clone 11-26c2a	BD Biosciences	564273	9066574
Anti-mouse IgD	APC-Cy7, clone 11-26c2a	Biolegend	405716	B178744
Anti-mouse IgG1	APC, clone X56	BD Biosciences	550874	58836
Anti-mouse IgG2a	PE, clone RMG2a-62	Biolegend	407108	B300880
Anti-mouse IgG2b	PE, clone RMG2b-1	Biolegend	406708	B321175
Anti-mouse IgM	BV605, clone RMM-1	Biolegend	406523	B323099
Anti-mouse IgM	PerCP-efluor7F10, clone II/41	eBioscience	46-5790-82	1995344
Anti-mouse PDL2	BV510, clone TY25	BD Biosciences	740194	9268285
Anti-mouse PDL2	Biotin, clone TY25	eBioscience	13-5986-82	B230798
Anti-mouse CD4	PerCP, clone RM4-5	Biolegend	100537	B283420

Anti-mouse PD1	APC, clone RMP1-30	Biolegend	109111	N309396
Anti-mouse CXCR5	BV421, clone L138D7	Biolegend	145511	B308119
Anti-mouse CD44	BV650, clone IM7	BD Bioscience	740455	315164
Anti-mouse CD62L	AlexaFluor 700, clone MEL-14	Biolegend	104426	B268247
Anti-mouse CD69	FITC, clone H1.2F3	BD Biosciences	01504D	M044628
**Other staining reagents**				
Live/Dead staining kit	Excitation 405nm	Invitrogen	L34957	2145008
Live/Dead staining kit	Excitation UV 350nm	Invitrogen	L34961	2264488
Streptavidin	BV785	Biolegend	405249	B230798
Streptavidin	PE-Cy7	Sony	2626030	178342
Streptavidin	PE	BD Biosciences	554061	38173
1X RBC Lysis Buffer		eBioscience	00-4333-57	
**Cell purification kits**				
Pan B Cell Isolation Kit II, mouse		Miltenyi	130-104-443	
Germinal Center B Cell (PNA) MicroBead Kit, mouse		Miltenyi	130-110-479	
$CD4^+$ T Cell Isolation Kit, mouse		Miltenyi	130-104-454	

Supplementary figure 1

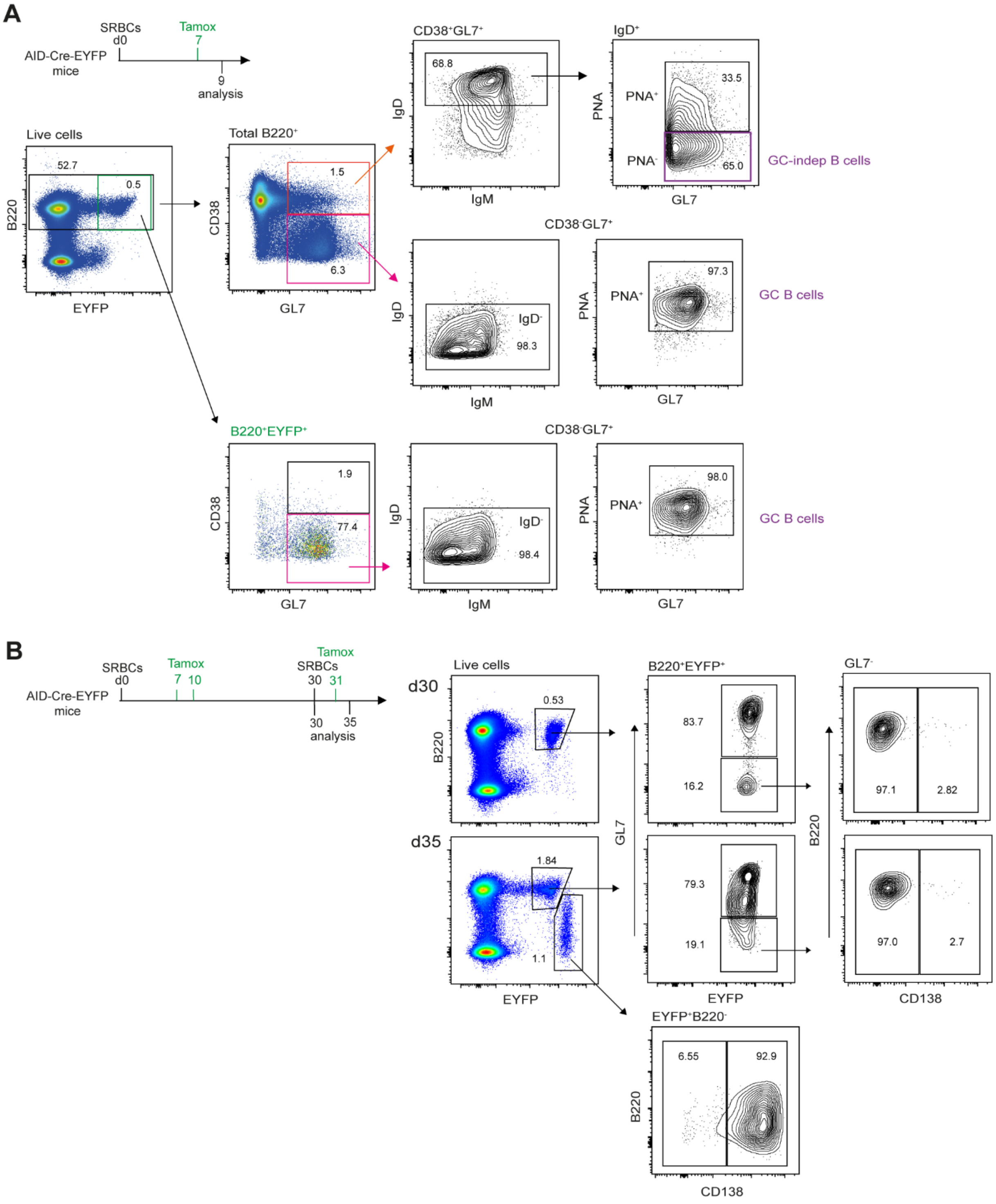

Supplementary figure 1. The AID-Cre-EYFP fate mapping model does not mark GC-independent, activated B cells. (A) AID-Cre-EYFP mice were injected with SRBCs (d0), received tamoxifen gavage on d7 and spleens were analyzed on d9. A representative flow cytometry plot (out of 4 analyzed mice) shows CD38 versus GL7 labeling on total B220$^+$ B cells. Germinal center (GC)-independent B cells can be identified among the CD38$^+$GL7$^+$ and IgD$^+$ cell population. Those cells are largely PNA$^-$ cells. CD38$^-$GL7$^+$ cells represent GC B cells which are IgD$^-$ and PNA$^+$ cells. The EYFP$^+$ labeled GL7$^+$ B cell population consists almost entirely of CD38$^-$, IgD$^-$ and PNA$^+$ GC B cells. **(B)** AID-Cre-EYFP mice were injected with SRBCs (d0) and received tamoxifen gavage on d7 and 10. Mice that were boosted on

d30 received tamoxifen on d31. Spleen were analyzed on d30 (before boost) and d35 (5 days after boost). Representative flow cytometry analyses (one out of three for each time point) show the percentage of $CD138^+$ cells into $EYFP^+B220^+GL7^-$ gated memory B cells (MBCs). The percentage of $CD138^+$ cells into the $EYFP^+B220^-$ population is shown for the staining on d35.

Supplementary figure 2

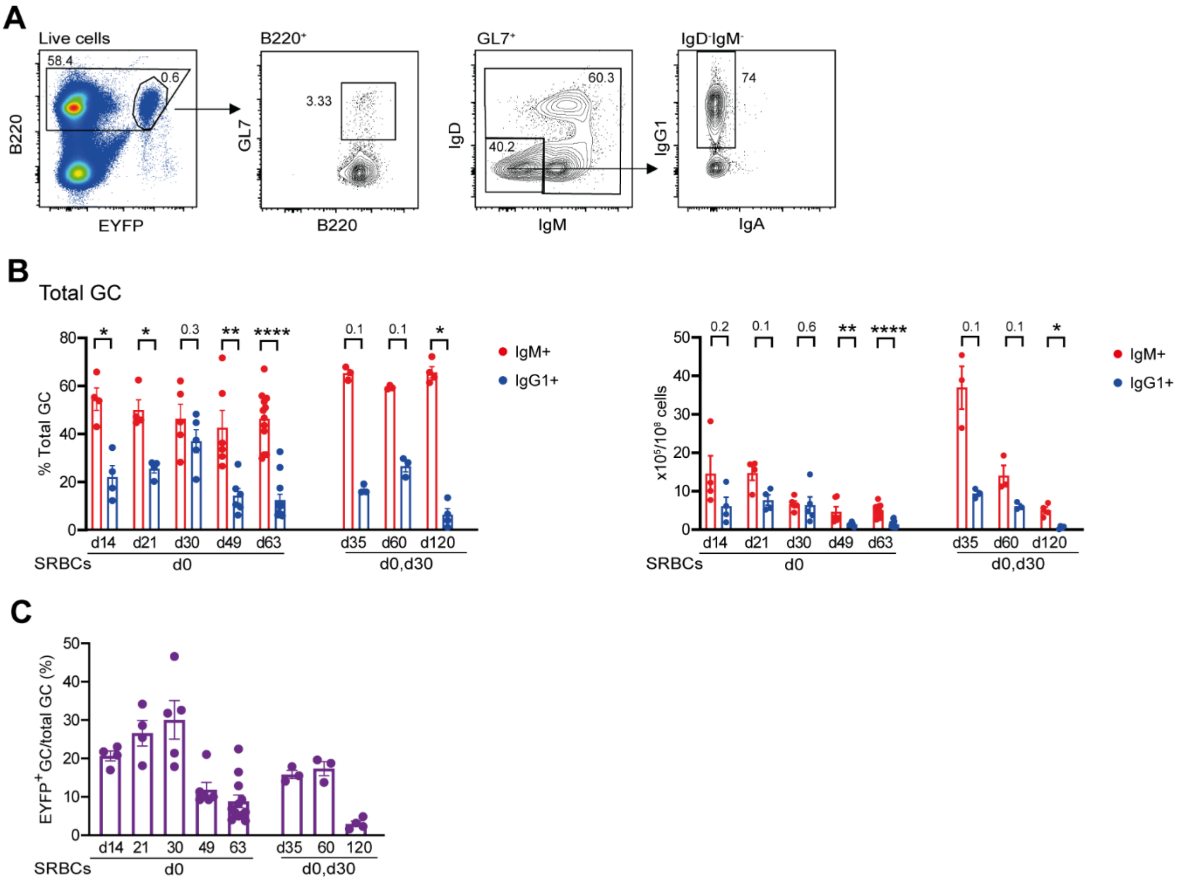

Supplementary figure 2. Characteristics of the total GC response after SRBC immunization. AID-Cre-EYFP mice were primed i.p. with SRBCs and, where indicated, received boost injections on day 30. Tamoxifen was administrated on d7,10 and 31 (when a boost on d30 was performed). Analyses were done on splenocytes at different time points: d14, 21, 30, 35, 49, 60, 63, 120. **(A)** Representative flow cytometry gating strategy to identify GC B cells among $B220^+$ cells and IgM/IgD, IgG1 vs. IgA isotype subclass distribution. **(B)** Distribution of total GCs B cells between IgM and IgG1 isotype expression. **(C)** Fractions of $EYFP^+$ within total GC B cells. p values>0.05 are indicated on panel **B**; *p<0.05, **p<0.01, ****p<0.0001.

Supplementary figure 3

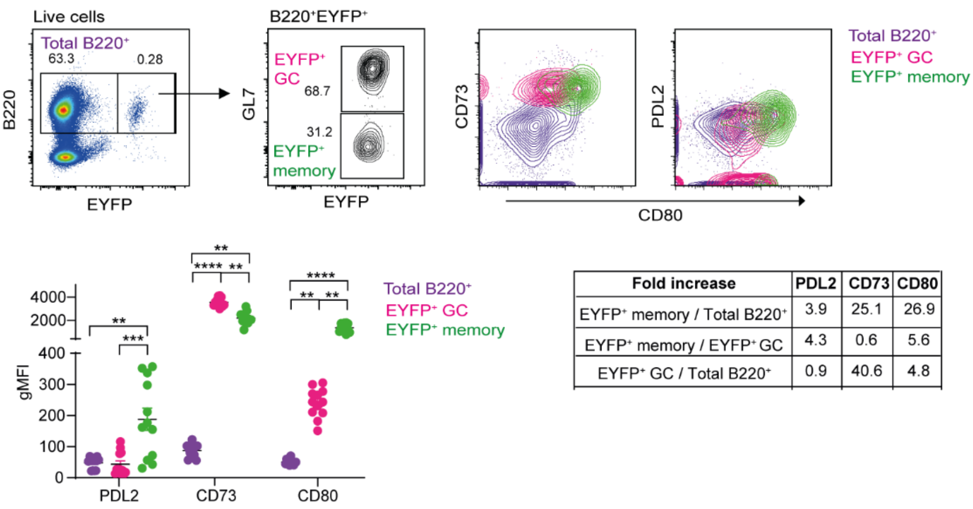

Fold increase	PDL2	CD73	CD80
EYFP+ memory / Total B220+	3.9	25.1	26.9
EYFP+ memory / EYFP+ GC	4.3	0.6	5.6
EYFP+ GC / Total B220+	0.9	40.6	4.8

Supplementary figure 3. Different expression of PDL2, CD73 and CD80 on MBCs compared to the total B cell population and GC B cells. AID-Cre-EYFP mice immunized with SRBCs received three doses of tamoxifen on d6,10,14. Spleens were analyzed 9 weeks after prime injection to characterize maturation markers expressed by $EYFP^{+}$ MBCs. The expression of CD73, CD80 and PDL2 was assessed on total $B220^{+}$ cells, $EYFP^{+}GL7^{+}$ GC and $EYFP^{+}GL7^{-}$ memory cells. A representative flow cytometry profile shows the three B cell populations. gMFI of PDL2, CD73 and CD80 are indicated in the scatter plot. Fold increases of the gMFI mean values are reported in the table. **p<0.01, ***p<0.001, ****p<0.0001.

Supplementary figure 4

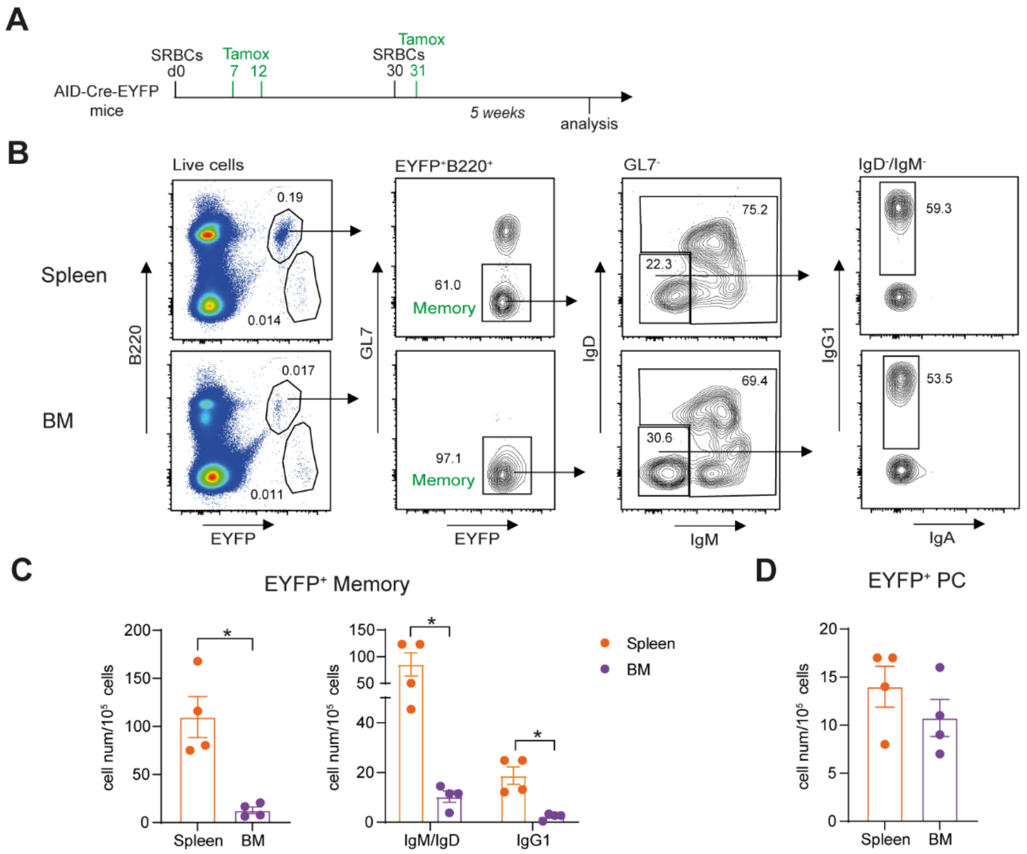

Supplementary figure 4. The spleen is the major anatomic reservoir of MBCs in the SRBCs immunization setting. (A) AID-Cre-EYFP mice were injected twice with SRBCs (d0 and 30) and received tamoxifen ingestion on d7,12 and 31. Mice were analyzed 5 weeks after boost injection. **(B)** A representative flow cytometry analysis of spleen and bone marrow (BM) cells is shown. MBCs were identified as GL7^{-}EYFP^{+}B220^{+} cells and their IgD/IgM and IgG1/IgA isotype profile was also determined. **(C)** EYFP^{+} MBC counts and isotype distribution (relative to 10^5 live cells) are shown in the plots for both spleen and BM. **(D)** EYFP^{+} plasma cells (PCs), represented by B220-EYFP^{+} cells were also enumerated. Each point in the graphs represents an individual mouse. Means (±SEM) are shown. Mann Whitney test was used to compare the conditions analyzed. *$p<0.05$.

Supplementary figure 5

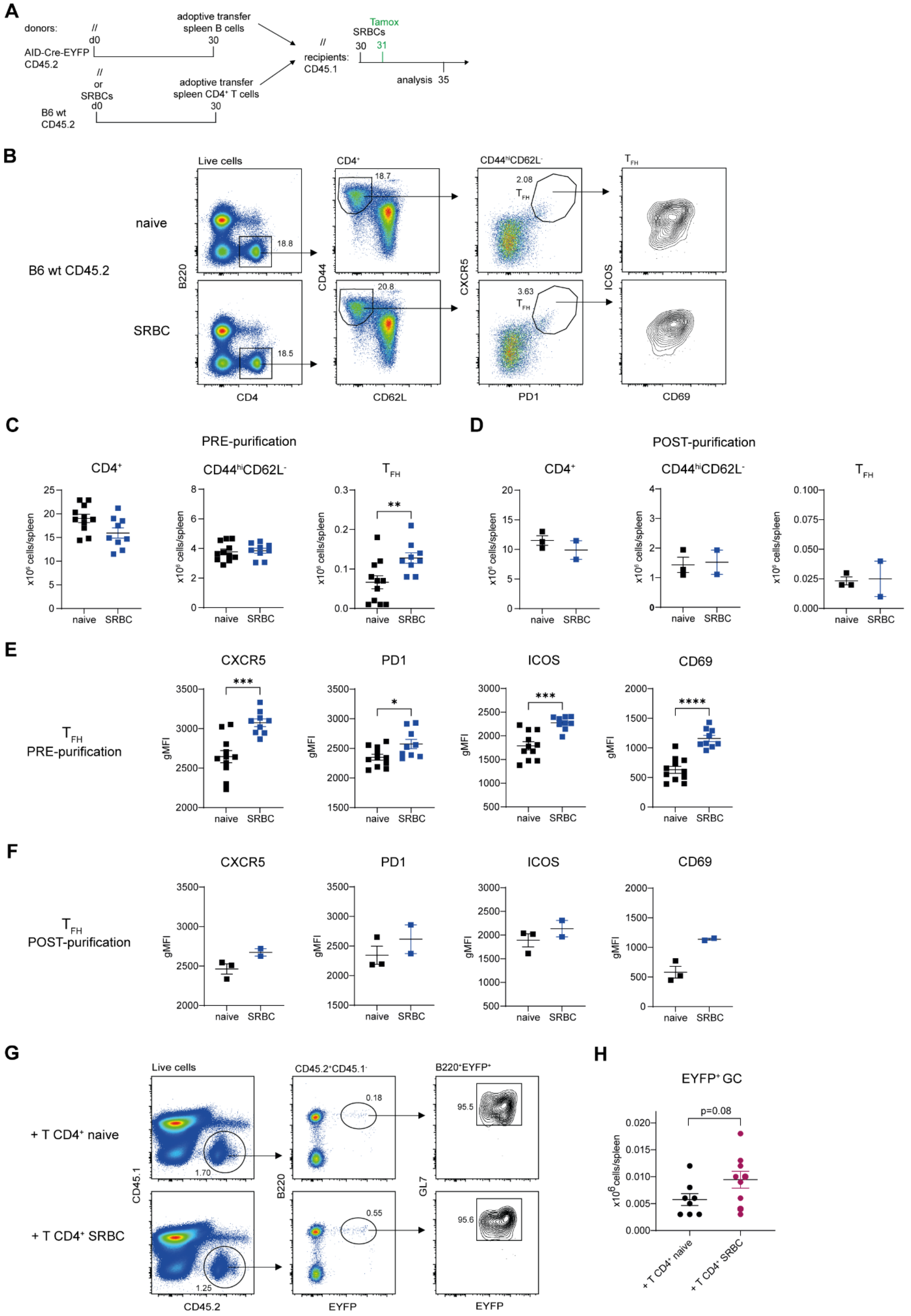

Supplementary figure 5. Memory T cells participate to the mobilization of naive B cells upon SRBC immunization. (A) 10 x10^6 total purified splenic B cells from naive (//) AID-Cre-EYFP donor mice were adoptively transferred together with 10 x10^6 purified CD4$^+$ T cells from naive (//) or SRBC-primed (d30) B6 wt CD45.2 mice into naive wt CD45.1 recipient mice. Host mice were injected with SRBCs 2 hours after adoptive cell transfer and received tamoxifen the following day. Spleens from recipient mice were analyzed by flow cytometry 5 days after SRBC challenge. **(B)** CXCR5^{hi}PD1^{hi} T_{FH} cells were identified within CD44^{hi}CD62L$^-$ activated CD4$^+$ T cells. The expression of CD69 and ICOS was further evaluated on the T_{FH} cell subset. Cell numbers of the aforementioned T cell subsets were calculated from each single spleen before cell purification **(C)** and from CD4$^+$ T cells purified from pooled spleen **(D)**. gMFI of CXCR5, PD1, ICOS, CD69 on T_{FH} cells are shown before **(E)** and after **(F)** T cell purification. (**G, H**) CD45.1$^-$CD45.2$^+$ donor cells from recipient spleens were gated into B220$^+$EYFP$^+$ cells and then EYFP$^+$GL7$^+$ GC counts were determined. Each point in panel **H** represents an individual mouse from at least two independent experiments. Means (±SEM) are shown. Mann Whitney test was used to compare between them two groups of mice. *$p<0.05$, ** $p<0.01$, ***$p<0.001$, ****$p<0.0001$. The exact p value is shown for panel **H**.

Supplementary figure 6

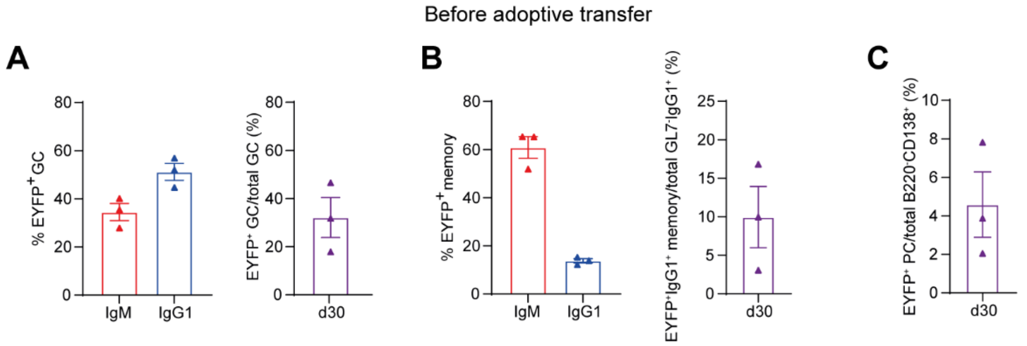

Supplementary figure 6. EYFP$^+$ cell profile 30 days after SRBC immunization (related to figure 2D). AID-Cre-EYFP mice were injected with SRBCs and received tamoxifen ingestion on d6,10,14. Splenic purified B cells were analyzed before adoptive transfer. **(A)** Distribution of IgM$^+$ and IgG1$^+$ cells among EYFP$^+$ GC B cells and EYFP$^+$ GC cell fraction calculated on total GC B cells are shown in the plots. **(B)** Distribution of IgM$^+$ and IgG1$^+$ cells among EYFP$^+$ MBCs and EYFP$^+$IgG1$^+$ MBC fractions calculated on total GL7$^-$IgG1$^+$ cells are shown in the graphs. **(C)** EYFP$^+$ PC fraction calculated on total B220$^-$CD138$^+$ cells are shown in the graphs.

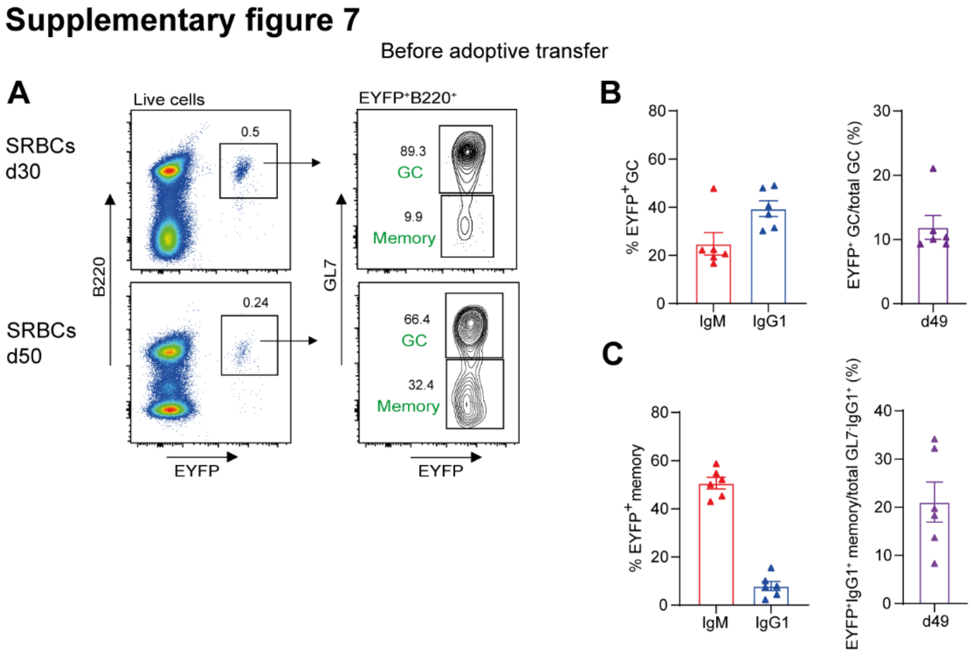

Supplementary figure 7. Increase of the MBC fraction among B220$^+$EYFP$^+$ cells between day 30 and d50 after SRBCs immunization. AID-Cre-EYFP mice were injected with SRBCs and received tamoxifen ingestion on d6,10,14. **(A)** Representative flow cytometry profiles, obtained 30 and 50 days after SRBCs prime, show the amount of B220$^+$EYFP$^+$ GC and MBCs, respectively identified as GL7$^+$ and GL7$^-$ cells. **(B)** Distribution of IgM$^+$ and IgG1$^+$ cells among EYFP$^+$ GC B cells at day 49 after priming and EYFP$^+$ cell fraction calculated on total GC B cells are shown in the plots. **(C)** Distribution of IgM$^+$ and IgG1$^+$ cells among EYFP$^+$ MBCs at day 49 after priming and EYFP$^+$IgG1$^+$ MBC cell fraction calculated on total GL7$^-$IgG1$^+$ cells are shown in the plots.

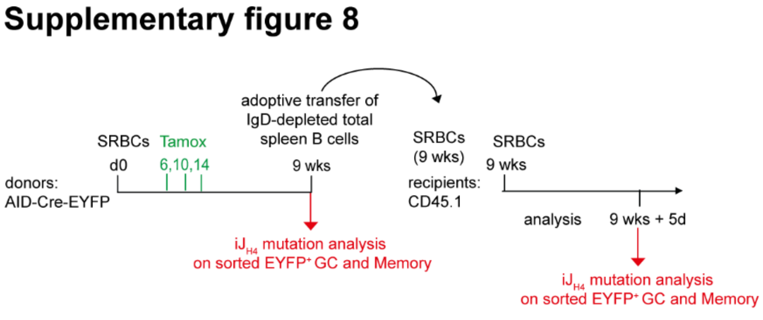

Supplementary figure 8. Experimental setting used for the mutation analysis of rearranged intronic J_{H4} sequences from EYFP$^+$ GC and memory sorted cells. iJH_4 mutation analysis was performed on EYFP$^+$ GC and MBCs that were sorted from AID-Cre-EYFP SRBCs-primed (9 weeks) donor mice that received tamoxifen on d6,10,14 and from SRBCs-primed (9 weeks) congenic wt CD45.1 recipient mice that received IgD-depleted B cells from the same AID-Cre-EYFP, SRBC-primed mice and SRBC challenge after cell transfer (cell sorting was performed 5 days later).

Supplementary figure 9

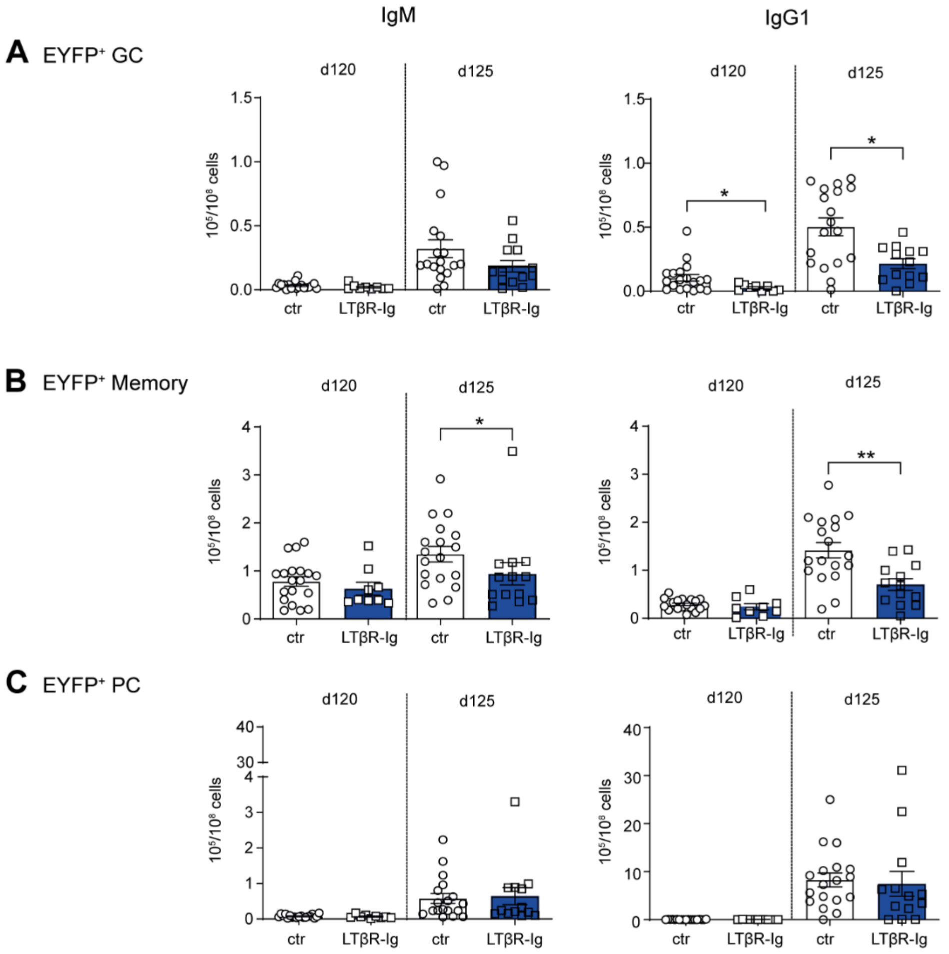

Supplementary figure 9. IgM+ and IgG1+ cell counts of EYFP+ GC, memory and PCs. from LTβR-Ig treated mice. AID-Cre-EYFP mice were immunized twice with SRBCs (d0 and 30) and received tamoxifen upon prime and boost (d7,12,31). 3 weeks after boost, a group of mice was i.v. injected with LTβR-Ig fusion protein 5 times 3 days apart (see Figure 4A). Spleen cells from treated mice and control mice (that did not receive any treatment) were analyzed by flow cytometry on d120 or five days after a tertiary SRBC injection performed on d120. Counts of IgM+ and IgG1+ EYFP+ GC **(A)**, memory **(B)** and PC **(C)** are shown for control and treated mice on both d120 and 125. Each point in the graphs represents an individual mouse from at least two independent experiments. Means (±SEM) are shown. Mann Whitney test was used to compare control and treated mice at the two time points analyzed. *p<0.05, **p<0.01. ctr=control mice.
